# Dietary stigmastane-type saponins as promising dual-target directed inhibitors of SARS-CoV-2 proteases: a structure-based screening†

**DOI:** 10.1039/d1ra05976a

**Published:** 2021-10-12

**Authors:** Oludare M. Ogunyemi, Gideon A. Gyebi, Ibrahim M. Ibrahim, Charles O. Olaiya, Joshua O. Ocheje, Modupe M. Fabusiwa, Joseph O. Adebayo

**Affiliations:** Human Nutraceuticals and Bioinformatics Research Unit, Department of Biochemistry, Salem University Lokoja Nigeria; Department of Biochemistry, Faculty of Science and Technology, Bingham University P.M.B 005, Karu Nasarawa Nigeria gideonagyebi@gmail.com gyebi.gideon@binghamuni.edu.ng +234-7063983652; Department of Biophysics, Faculty of Sciences, Cairo University Giza Egypt; Nutritional and Industrial Biochemistry Unit, Department of Biochemistry, University of Ibadan Nigeria; Department of Pure and Industrial Chemistry, Nnamdi Azikiwe University Akwa Nigeria; Department of Biochemistry, Faculty of Life Sciences, University of Ilorin Ilorin Nigeria

## Abstract

Despite the development of COVID-19 vaccines, at present, there is still no approved antiviral drug against the pandemic. The SARS-CoV-2 3-chymotrypsin-like proteases (S-3CLpro) and papain-like protease (S-PLpro) are essential for the viral proliferation cycle, hence attractive drug targets. Plant-based dietary components that have been extensively reported for antiviral activities may serve as cheap sources of preventive nutraceuticals and/or antiviral drugs. A custom-made library of 176 phytochemicals from five West African antiviral culinary herbs was screened for potential dual-target-directed inhibitors of S-3CLpro and S-PLpro *in silico*. The docking analysis revealed fifteen steroidal saponins (FSS) from *Vernonia amygdalina* with the highest binding tendency for the active sites of S-3CLpro and S-PLpro. In an optimized docking analysis, the FSS were further docked against four equilibrated conformers of the S-3CLpro and S-PLpro. Three stigmastane-type steroidal saponins (vernonioside A2, vernonioside A4 and vernonioside D2) were revealed as the lead compounds. These compounds interacted with the catalytic residues of both S-3CLpro and S-PLpro, thereby exhibiting dual inhibitory potential against these SARS-CoV-2 cysteine proteases. The binding free energy calculations further corroborated the static and optimized docking analysis. The complexed proteases with these promising phytochemicals were stable during a full atomistic MD simulation while the phytochemicals exhibited favourable physicochemical and ADMET properties, hence, recommended as promising inhibitors of SARS-CoV-2 cysteine proteases.

## Abstract 2

*Vernonia amygdalina* derived phytochemicals as potential dual-target directed inhibitors of SARS-CoV-2 proteases from computational study perspective.

## Graphical abstract

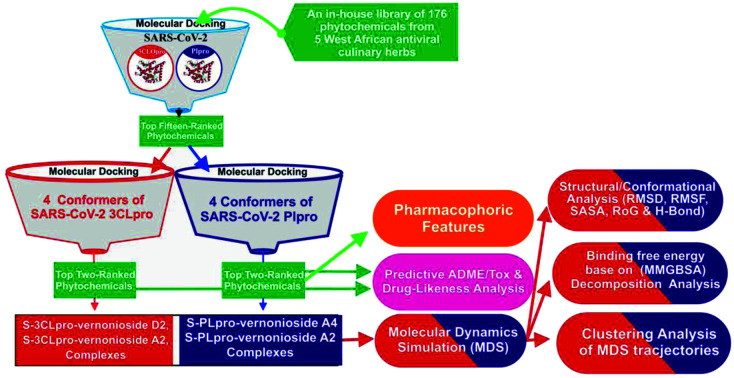

## 1 Introduction

The coronavirus disease 19 (COVID-19) pandemic, caused by the virus severe acute respiratory syndrome corona virus 2 (SARS-CoV-2), is one of the most historical formidable global health risks with its severe adverse effects on national economies, public health, educational system, social life as well as personal lives. Consequently, substantial multidisciplinary and collaborative research efforts have been geared towards the epidemiology, diagnosis, and therapy of COVID-19. Drug repurposing and search for new antiviral formulations that target pathogenesis and replication cycle of the COVID-19 virus is a major area that has attracted attention. In this direction, key proteins involved in the viral replication process and infectivity that are highly conserved or contain conserved motifs among coronaviruses, present possible targets for anti-coronavirus therapeutic development in COVID-19. The SARS-CoV-2 and related coronaviruses possess a positive sense, single-stranded large RNA genome, which is about 30 kb size.^1,2^ Upon attachment of the virus to human host cell at the infection stage, it releases its RNA genome into the cytoplasm and thereafter, a number of molecular processes are activated to produce the new RNA of the virus as well as the proteins that form its envelope. The conserved SARS-CoV-2 genome contains a huge replicase gene, encoding 16 nonstructural proteins (NSPs),^3^ preceding structural and accent genes.^2,4^ The nonstructural proteins (nsps) are known to possess multiple enzymatic activities.^3^ Two-thirds of the genome from the 5′ end consists of two open reading frames (ORF1a and ORF1b) which are translated into the two polyproteins (pp1a and pp1ab).

The cysteine proteases, the papain-like proteases (PLpro) and 3-chymotrypsin-like protease (3CLpro) are essential for viral replication as they auto-proteolytically cleave the newly generated viral polyproteins chain to release the NSPs,^5^ which are pivotal to viral replication and, hence, more infections.^6–8^ Thus, they are attractive drug target for developing anti-SARS-CoV-2 drugs in COVID-19. Among the 16 NSPs 11 are generated by the 3CLpro, hence is known as the main protease. This enzyme contain a highly conserved catalytic dyad comprising amino acid residues His-41 and Cys-145.^9^ The 3CLpro owes its uniqueness to the occurrence of an unconventional catalytic cysteine residue as compared to other chymotrypsin like enzymes and other Ser (or Cys) hydrolases.^10^ In particular, the SARS-CoV-2 3CLpro consist of a catalytic Cys145–His41 dyad in the place of a canonical Ser(Cys)–His–Asp(Glu) triad.^11^ Structurally, these residues are buried in a surface pocket (active site cavity) of the protein^12^ and the His-41 and Cys-145 are at a distance of 3.8 Å.^10,12,13^ The enzymatic activity of the second cysteine protease, PLpro reside in active site containing the catalytic triad formed by CYS^111^, HIS^272^ and ASP^286^ residues. These key residues are involved in the cleavage of the protein substrate pp1a/ab polyprotein to release nsp1, nsp2 and nsp3. Thus, inhibition of these important cysteine proteases (3CL^pro^ and PL^pro^) may significantly impede the viral machinery and overall viral infection rate. Therefore, both enzymes are attractive targets for the development of antiviral drugs directed against SARS-CoV and other coronavirus infections. Furthermore, exploiting the catalytic similarities between 3CLpro and PLpro as a common therapeutic target, single inhibitors with dual inhibitory activity against both proteases might help in the development a dual-target directed treatment regimen for COVID-19. The potentials of dual-target-directed inhibitors as antivirals have been reported previously for viruses such as dengue virus,^14^ hepatitis B virus,^15^ and SARS-CoV-2.^8^

During the current pandemic, the urgent need for prevention and therapy in COVID-19 especially in the developing countries has led to the use of several tropical food herbs and culinary spices which have been documented for the effective management of cold, flu and all other common viral symptoms.^16^ Several African culinary herbs and spices have also been extensively reported for antiviral activities; thus, are increasingly becoming vegetal resources for therapeutic and pharmacological agents in drug discovery, design and development. Such antiviral food plants include: African tea leaf (*Vernonia amygdalina* Del.),^17,18^ African basil (*Ocimum gratissimum*)^19,20^ and *Aframomum melegueta* and *Piper guineense*.^21^ These dietary components as well as their isolated compounds might serve as functional foods, nutraceuticals and potent alternative medicines for COVID-19. Previous reports have revealed that, food plant-derived phytochemicals may target SARS-CoV-2 replication machinery^22,23^ and other important proteins.^24,25^ Many of such phytochemicals derived from some Indian spices as inhibitors of SARS-CoV-2 main protease have been tested and reported recently.^26^ The aim of this study was to identify promising inhibitors of these proteases from some West African food herbs and spices through structure-based virtual screening.

## 2 Experimental

### 2.1 Protein structure retrieval and preparation for molecular docking

The recently deposited three-dimensional (3D) SARS-CoV-2 3CL^pro^ (S-3CL^pro^) (PDB ID 6Y2E) and SARS-CoV-2 PL^pro^ (S-PL^pro^) (PDB ID 6W9C) structures were downloaded from the Protein Data Bank (http://www.rcsb.org). The structures of the downloaded the proteins were prepared by removing existing ligands and water molecules. The missing hydrogen atoms were added while the Kollamn charge were added as the partial atomic charge in MGL-AutoDockTools (ADT, v1.5.6).^27^ The well-ordered scheme was repeated for each protein target and subsequently saved as dockable pdbqt format for molecular docking.

### 2.2 Ligand structure preparation

Previously reported 176 compounds from the African tea leaf (*Vernonia amygdalina*), African basil (*Ocimum gratissimum*), *Tetrapleura tetraptera*, *Aframomum melegueta* and *Piper guineense* were compiled from several literatures including review articles, articles involving identification and isolation for constituent phytochemicals. The Structure Data Format (SDF) of these phytochemicals as reference inhibitors (alpha-ketoamide, ritonavir and GRL0617) were retrieved from the PubChem database (http://www.pubchem.ncbi.nlm.nih.gov), while compounds that were not available on the database were drawn with ChemDraw version 19. All structures were converted to mol2 chemical format using Open babel,^28^ polar hydrogen charges of the Gasteiger-type were assigned to atoms, while the non-polar hydrogen molecules were merged with the carbons. The ligands were then converted to the dockable PDBQT format using AutoDock Tools for *in silico* analysis.

### 2.3 Molecular docking studies

#### 2.3.1 Active site targeted molecular docking of phytochemicals

The 176 phytochemicals and reference inhibitors were docked into the active site of S-3CLpro through AutoDock Vina in PyRx 0.8.^29^ Based on the binding energies, interaction with the catalytic residues and binding poses a hit-list of 64 phytochemicals was defined with binding energies comparable to the reference inhibitor (ritonavir). The 64 hit phytochemicals were subsequently docked into the active sites of S-PL^pro^ towards identify multi-target-directed compounds. OpenBabel^28^ incorporated into PyRx 0.8 was employed for importing and energy minimization of the ligand structures. In the software, the Universal Force Field (UFF) was used as the energy minimization parameter and conjugate gradient descent as the optimization algorithm. The parameters of the regions enclosing the active sites of the three proteins as defined by the grid boxes are presented in Table 1. All the other parameters were kept as default. Discovery Studio Visualizer version 16 was used to view the detected molecular interactions.

**Table 1:** Binding site coordinates of SARS-CoV-2 protease

Dimensions	SARS-CoV-2 3CL^pro^ (Å)	SARS-CoV-2 PL^pro^ (Å)
Center_*x*	−16.26	−1.406
Center_*y*	−27.23	−13.00
Center_*z*	2.92	−8.26
Size *x*	21.88	24.53
Size *y*	25.96	14.84
Size *z*	27.88	19.36

### 2.4 Molecular dynamics simulation

#### 2.4.1 Molecular dynamics simulation of unbound SARS-CoV-2 3CL^pro^ and PL^pro^

The structure of SARS-CoV-2 3CL^pro^ and PLpro were retrieved from the Protein Data Bank with codes 6Y84 and 6W9C.^30,31^ The apo (unbound) protein structures of both proteases were submitted for a 100 ns atomistic molecular dynamics (MD) simulation production run using the NVT ensemble (normal volume and temperature with a constant number of atoms). Prior to the production run, the systems were minimization for 10 000 steps through a conjugate gradient algorithm. CHARMM 36 force field was used on the Nanoscale Molecular Dynamics (NAMD 2.13) software.^32,33^ Preparation of the input files and analysis of the output trajectories was performed using the Visualizing Molecular Dynamics (VMD 1.9.3) software.^34^ Water boxes were added to the proteins subsequent to addition of the missing hydrogen atoms and removal of any ligand. For the periodic boundary to be applied, TIP3P water model was used to resemble the added water box, with 10 Å padding.^35^ The pressure of the system was controlled at 1.01325 bar using the Nose–Hoover–Langevin piston. In contrast, the Langevin dynamics was employed to control the system's temperature at physiological value. The parameters such as temperature, salt concentration, and pH were set at the physiological values (310 K, 0.154 M NaCl and 7.0 respectively) during the simulation period. The time step was set at its default two fs with SHAKE approximation in action.

#### 2.4.2 Clustering of molecular dynamic trajectories of unbound SARS-CoV-2 3CL^pro^ and PL^pro^

TTClust V 4.9.0 (ref. 36) was used to cluster the MD simulation trajectories for unbound S-3CLpro and S-PLpro. Each system was clustered automatically using TTClust python package, which utilize the elbow method to determine the optimal number of clusters, and finally producing a representative frame for each cluster. A representative conformation was selected from each cluster and used in the in-depth docking experiment.

### 2.5 Docking analysis of the lead phytochemicals to conformers of the receptors

The fifteen hit phytocompounds (FHP) from the initial docking experiments to both protease and the positive controls (α-ketoamide, ritonavir and GRL0617) were docked to four different representative conformers of S-3CLpro and S-PLpro after the cluster analysis of the MDS trajectories using AutoDock Vina software.^29,37^ The Universal Force Field (UFF) was utilized to optimize it using the steepest descent algorithm.^38–40^ PyMOL 2.4 software and the Protein–Ligand Interaction Profiler (PLIP) web server were employed to analyze the docking complexes.^41^

### 2.6 Molecular dynamics simulation, structural stability and thermodynamic parameters analysis of lead complexes

The topmost four complexes from the docking analysis of the phytocompounds with the two SARS-CoV-2 proteases *viz*: S-3CL^pro^-vernonioside D2, S-3CL^pro^-vernonioside A2, S-PL^pro^-vernonioside A4 and S-PL^pro^-vernonioside A2 were selected for Molecular Dynamic Simulation (MDS) using NAMD 2.13. The MDS files were generated using CHARMM-GUI.^42–44^ The salt concentration and temperature were adjusted to 0.154 NaCl and 310 K, respectively, so as to mimic the physiological conditions. Before running the production run in which the two 3CLpro complexes were allowed to explore their conformational spaces for 50 nanoseconds while the PLpro complexes were allowed to run for 35 ns, in a constant number of atoms, constant volume, and constant temperature (NVT) ensemble using a conjugate gradient algorithm the system was minimized for 10 000 steps then equilibrated in a constant number of atoms, constant pressure, and constant temperature (NPT) ensemble for one ns. The pressure was set to atmospheric pressure (1.01325) bar and controlled by the Nose–Hoover–Langevin piston, while the temperature was controlled by Langevin dynamics. The force field used was the CHARMM36 force field. The trajectories were saved each 0.1 ns. VMD^45^ was used to perform the analysis of the trajectories by calculating the Root Mean Square Deviation (RMSD), Root Mean Square Fluctuation (RMSF), Surface Accessible Surface Area (SASA), Radius of Gyration (RoG), and hydrogen bonds (H-bonds).

#### 2.6.1 Binding free energy calculations

Binding free energy using Molecular Mechanics Generalized Born Surface Area (MMGBSA) implemented in MMPBSA.py in Ambertools 17 package was calculated for the two lead complexes for the SARS-CoV-2 proteases. Saltcon variable was set to 0154 M and the method of generalized Born (igb) was set to 5. In addition, decomposition of the free energy was obtained to determine the contribution of each amino acid toward the binding.^46,47^

#### 2.6.2 Clustering of molecular dynamic trajectories of lead-SARS-CoV-2 proteases complexes

TTClust V 4.9.0 (ref. 36) was used to cluster the MD simulation trajectories for the four lead complexes for the SARS-CoV-2 proteases by finding the optimum number of clusters using the elbow method. Protein–Ligand Interaction Profiler (PLIP) was used to determine the number and types of interactions between the phytochemicals and the proteases.^48^ Amino acids numbers in all of the analyses starts from number 1 until the end of the sequence without any gaps.

### 2.7 *In silico* physicochemical properties and ADMET study

The lead phytochemicals that were selected based on their binding affinity and docked poses with the four different equilibrated conformers were subjected to descriptors of drug-likeness and ADMET filtering analysis. Five drug-likeness filters (Lipinski, Veber, Ghose, Egan and Muegge) were performed on the SwissADME (http://www.swissadme.ch/index.php) webserver.,^49^ while the predicted Absorption–Distribution–Metabolism–Excretion–Toxicity (ADMET) analysis was performed on the SuperPred webserver (http://lmmd.ecust.edu.cn/admetsar1/predict/).^50^ To this end, the canonical SMILES and SDF format of the compounds were retrieved from PubChem Database or retrieved from ChemDraw.

### 2.8 Identification of pharmacophoric features of lead phytochemicals

The online webserver PharmaGist (https://bioinfo3d.cs.tau.ac.il/PharmaGist/)^51^ and Zincpharmer (http://zincpharmer.csb.pitt.edu/pharmer.html)^52^ were employ to generate the pharmacophore model for the lead phytochemicals. Based on the physicochemical features and pharmacophore score, the best pharmacophore was selected and downloaded in mol2 format for further analysis. The three lead phytochemicals were flexibly aligned to generate the merged pharmacophore model. All the shared features of the three lead phytochemicals were considered during model generation. The hydrogen bond acceptor (HBA), hydrogen bond donor (HBD), hydrophobic (HYD), aromatic ring (AR), positive ionizable (PI), negative ionizable (N.I.) features were the selected features of the model.

## 3 Results and discussion

### 3.1 Screening of phytochemicals against the active site of SARS-CoV-2 3CL^pro^ and PL^pro^

**Fig. 1:**
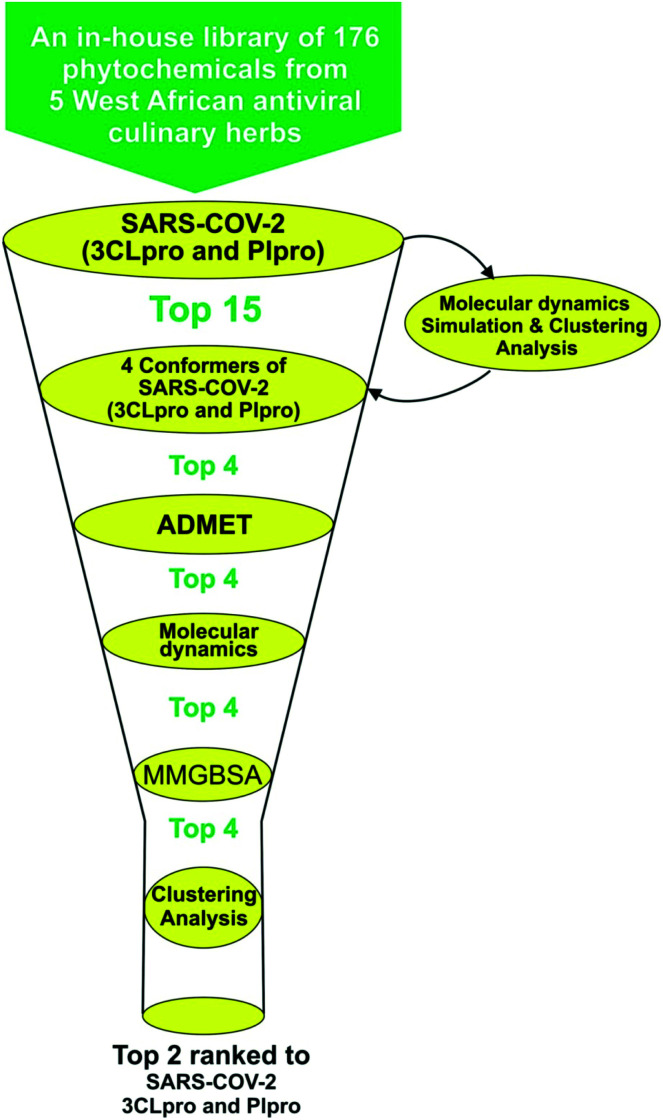
Flow chat for the virtual-screening of 176 phytochemicals from 5 West African antiviral culinary herbs.

Structure-based virtual screening tools such as molecular docking analysis are used to predict the best mode of interaction between a ligand and a receptor. This technique has been used widely to identify potential inhibitors of SARS-CoV-2 replication.^23,53^ The results for screening the phytochemicals from selected 5 West African antiviral culinary herbs and spices against the S-3CL^pro^ alongside the reference inhibitors (α-ketoamide 13b, ritonavir and GRL0617) is represented in (Table S1 ESI†). A hit list of 64 phytochemicals with docking scores comparable but not lower than the reference compound (ritonavir −6.8 kcal mol^−1^) was further docked into the active regions of S-PLpro for multi-target binding analysis (Table S2 ESI†). From the results of the later analysis, a hit list of the 15 phytochemicals (Table 2) belonging to the class of stigmastane-type steroid saponins glucoside from *Vernonia amygdalina* were selected based on their orientation at the catalytic site, the interacting residues and binding affinities comparable to those of reference inhibitors, alpha-ketoamide 13b (−7.7 kcal mol^−1^) and GRL0617 (−6.7 kcal mol^−1^) for S-3CL^pro^ and S-PLpro respectively (Table 2). The binding energies recorded from docking analysis of the reference inhibitors to the S-3CL^pro^ and S-PL^pro^ is similar with those reported from other studies.^8^ From the hit list of 15 phytochemicals, the three lead phytochemicals for both proteases were found to be vernonioside A2, vernonioside D2 and vernonioside A4 (−8.6, −8.4 and −8.3 kcal mol^−1^) and vernonioside A4, vernonioside A2 and vernoniamyoside C (−7.2, −6.8 and −6.7 kcal mol^−1^) for S-3CL^pro^ and S-PL^pro^ respectively (Table 3). Fig. 1 showing the flow chat for the computational methods employed in the screening of the 176 phytochemicals compiled from 5 West African antiviral culinary herbs.

**Table 2:** Binding docking scores of 15 lead phytochemicals docked in the active sites of 3-chymotrypsin-like and papain-like proteases of SARS-CoV-2^a^

S/no.	Compounds	Class of Compounds	Binding energies kcal mol^−1^	
			3CL^pro^	PLPro
S1	α-Ketoamide 13b		−7.7	
S2	Ritonavir		−6.8	
S3	GRL0617			−6.7
1	Vernonioside A2	Steroidal saponins	**−8.6**	**−6.8**
2	Vernonioside D2	Steroidal saponins	**−8.4**	−6.3
3	Vernonioside A4	Steroidal saponins	**−8.3**	**−7.2**
4	Vernonioside B3	Steroidal saponins	−8.1	−6.1
5	Vernodalin	Steroidal saponins	−8.1	−5.9
6	Vernoniamyoside C	Steroidal saponins	−8.0	**−6.7**
7	Vernonioside D	Steroidal saponins	−8.0	−6.1
8	Vernonioside B2	Steroidal saponins	−8.0	−5.9
9	11,13-Dihydrovernodalin	Steroidal saponins	−8.0	−5.7
10	Vernomygdin	Steroidal saponins	−7.9	−5.3
11	Neoandrographolide	Steroidal saponins	−7.8	−6.7
12	Vernoniamyoside A	Steroidal saponins	−7.8	−6.2
13	Vernonioside A3	Steroidal saponins	−7.8	−6.2
14	Vernonioside A1	Steroidal saponins	−7.8	−6.2
15	Andrographoside	Steroidal saponins	−7.8	−6.2

a Figures in bold represent the three lead compounds in view of binding scores.

**Table 3:** Molecular interactions of the amino acid residues of the 3-chymotrypsin-like and papain-like proteases of SARS-CoV-2 with the three lead phytocompounds

Compounds	SARS-CoV-2 protein	Hydrogen bonds (bond distance Å)		Hydrophobic interaction		Other interactions	
		Numbers	Interacted residues	Numbers	Interacted residues	Numbers	Interacted residues
Alpha-ketoamide		7	**Glu** ^**166**^ (2.88, 2.95), His^172^ (3.57), Met^165^ (2.97), His^41^ (2.68), **Cys**^**145**^ (3.03), Gly^143^ (2.76)	2	Met^165^, **His**^**41**^	2	**Glu** ^**166**^ (2)
Ritonavir	3CLpro	4	**Cys** ^**145**^ (3.57), His^163^ (3.57), His^164^ (3.40), Pro^168^ (3.40)	5	**His** ^**41**^ (2), Pro^168^, Met^49^, **Cys**^**145**^, Met^49^		None
Vernonioside A2		6	Leu^167^ (2.81), **Glu**^**166**^ (2.77), Gly^170^ (3.00), Pro^168^ (3.40), Asn^142^ (3.35), Gly^143^ (2.06)	3	**Cys** ^**145**^, **His**^**41**^, Pro^168^		None
Vernonioside A4		4	**Glu** ^**166**^ (2.23), Ser^46^ (2.89), Cys^44^ (2.45), Thr^25^ (2.74)	3	**His** ^**41**^, Met^49^, Met^165^		
Vernonioside D2		5	Leu^141^ (2.81), **Cys**^**145**^ (3.03), **Glu**^**166**^ (2.88), His^163^ (3.57), Asn^142^ (3.35)	2	**His** ^**41**^, Met^41^		None
GRL0617	PLpro	5	Gln269 (2.14), Asp164 (2.16), Try268 (2.88)		Pro248, Pro248, Try264, Try268, Leu162, Asp164, Gln269		None
Vernonioside A4		5	Try268 (2.88), Pro248 (2) (2.88), Gln250 (2.88)	3	Pro248 (2), Pro247		None
Vernonioside A2		2	Asn267 (2.67), Tyr264 (2.34)	6	Leu162, Pro248 (3), Tyr264 (2)		
Vernoniamyoside C		7	Tyr264 (2) (2.34), Gln250 (2.88), Asn267 (2.67), Pro248 (2) (2.88), Asp164 (2.34)	4	Pro247, Tyr264, Pro248 (2)		

#### 3.1.1 Molecular interactions between the lead phytochemicals and the active regions of SARS-CoV-2 3CL^pro^ and PLpro

The catalytic dyad (His^41^ and Cys^145^) at the active of S-3CLpro is located between its domain I (consisting of residues number 8–101) and domain II (consisting of residues number 102–184).^31^ The monomer is completed by the connecting long loop (consisting of residues number 185–200) of domain II and domain III (consisting residues 201–303).^54^ The main enzymatic activity of 3CL^pro^ resides in its catalytic dyad,^55^ while that of S-PL^Pro^ resides in the catalytic triad which is formed by CYS^111^, HIS^272^ and ASP^286^.^56,57^ The other catalytic residues of S-PL^Pro^ include TRP^106^, GLY^256^, and LYS^274^.^58^ The deubiquitinating activity of S-PLpro have been reported to be associated with residue LEU^162^, ASP^164^, GLU^167^and TYR^264^.^59^ The other catalytic residues of S-3CLpro have been described by various reports.^60–62^ In this study, in a similar binding pattern as the reference inhibitors, the three lead phytochemicals to the SARS-CoV-2 proteases were stabilized by numerous non-covalent interactions in the active regions of the target protein (Table 3). Ritonavir, an anti-retroviral protease with inhibitory activity against S-3CL^pro^ (ref. 63) and α-ketoamides a broad-spectrum inhibitor of the main proteases of betacoronaviruses, alphacoronaviruses as well as the 3C proteases of enteroviruses^64^ were docked into the substrate binding pocket in similar pattern reported in previous studies.^31^

**Fig. 2:**
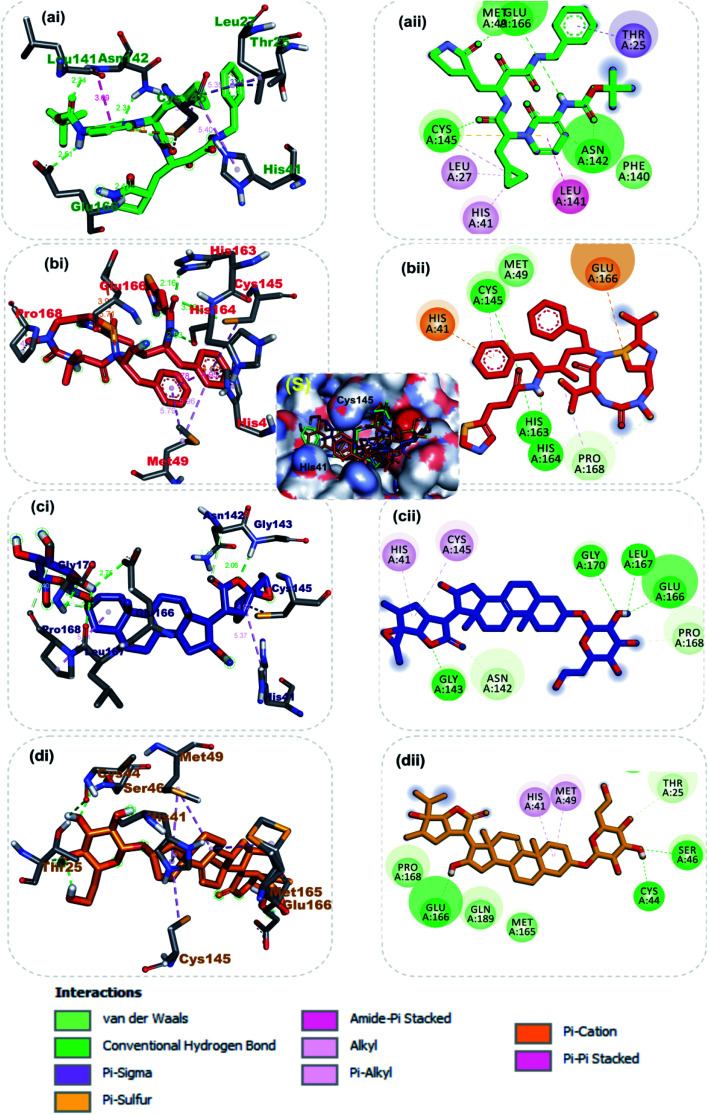
3D (i) and 2D (ii) amino acid interactions of phytochemicals and reference inhibitors in substrate binding cavity of SARS-CoV-2 3CLpro. (S) Surface representation. The ligands in stick representations are shown by colours (a) green: α-ketoamide (b) red: ritonavir C (c) blue: vernonioside A2 (d) gold: vernonioside A4.

The carbonyl group of the 1,3-thiazol-5-yl-methoxy-carbonyl-aminohexan-2-yl moiety of ritonavir accepted 3 hydrogen atoms from the 3-mercapto group of Cys145, 1*H*-imidazol-5-yl of His163 and His164 of S-3CLpro (Fig. 2b). Also, the amino group next to the carbonyl group of the same moiety formed a hydrogen bond to His165. The first phenyl ring formed a pi–cation and pi–alkyl hydrophobic interactions with His41 and Cys145 of the catalytic dyad while a strong attractive forces was observed between the side chain carbonyl group of Glu166 and sulphur of 2-propan-2-yl-1,3-thiazole group of ritonavir (Fig. 2b). From Fig. 2a α-ketoamide 13b an inhibitor of S-3CLpro was docked into the substrate-binding cleft located between domains I and II of the 3CL^pro^ as reported in an earlier study.^31^ The lead phytochemical (vernonioside A2) was docked into the substrate binding pocket of S-3CL^pro^ in a similar manner as the reference inhibitors (Fig. 2c). The 2-hydroxyl group or glycosyl unit vernonioside A2 accepted 2 hydrogen atoms from Gly143 and Gly170 while donating hydrogen to Leu167 to form 3 hydrogen bonds. Carbon-3 of the glycosyl units formed a carbon hydrogen bond with Pro168 of S-3CLpro. The carbonyl carbon at carbon-21 and the oxygen of tetrahydrofuran ring of the stigmastane aglycon formed 2 hydrogen bonds with Asn142 and Gly143, while the tetrahydrofuran formed 2pi–alkyl interacting with the catalytic dyad residues Cys145 and His41 S-3CLpro (Fig. 2c). The 3-hydroxyl of the glycosyl moiety of vernonioside A4 formed 2 hydrogen bonds with Cys44 and Ser46 of S-3CL^pro^ (Fig. 2d). The 16-hydroxyl group of the aglycon formed a hydrogen bond with Glu166, while the A ring of the stigmastane aglycon formed 2pi–alkyl interactions with Met49 and His41. The 3-hydroxyl of the glycosyl moiety formed 2 hydrogen bonds with His163 and Leu141 while the 2 and 6-hydroxyl group formed hydrogen bonds with Glu166 and Asn142 of the S-3CL^pro^. The A ring of vernonioside A4 formed an alkyl interaction with Met49, while a pi–sigma interactions was observed with the double bond between carbon-7 and carbon-8 (Fig. 2d).

Taken together, the co-crystalized compound α-ketoamide 13b, reference drug ritonavir, and the various high affinity vernonia-derived phytochemicals with S-3CLpro indicated several similarities in their interaction profile in the enzyme active site. They all interacted with the amino acids of the catalytic dyad (Cys145 and His41) and Glu166, which are required for S-3CLpro activity. Besides, it was also observed that, these compounds interacting with other neighboring amino acid residues. In particular, the hydrogen bonding interaction of Glu166 with vernonioside A2, vernonioside A4, and vernonioside D2 is very important, as the Glu166 is critical the dimerization of 3CLpro monomers.^13^ This dimer formed is important for its enzymatic activity of S-3CLpro. Thus, any interaction with Glu166 may cause formation of an inactive monomer which may ultimately interfere with the enzyme activity of 3CLpro.^12,13^ These, vernonia-derived phytocompounds had important interactions with the catalytic dyad (Cys145 and His41). During the proteolytic process of this enzyme, the catalytic dyad is known to be activated in the presence of the protein substrates which contain the amino acid sequence Leu–Gln↓Ser–Ala–Gly (↓ marks the cleavage site).^10^ In the inactive state, amino acids Cys145 and His41 in are separated by a distance of 3.8 Å as reviewed earlier.^10,12,13^ This distance between these amino acids is long enough such that, intermolecular hydrogen bond formation between them is prevented at physiological pH. Besides, the Cys145 is protonated at physiological pH and His41 is present in the neutral state. Substrate binding induces intramolecular proton transfer from Cys145 to His41 through attack of Cys145–sulfur onto the (C=O) of the substrate peptide.^11,12^ The protonated His41 is stabilized by the surrounding water molecules. The ability of the steroidal saponin structures to form a hydrogen bond and other interactions with both Cys145 and His41 residues or either with one of these catalytic dyad amino acid residues as observed in this study suggests their inhibitory potential against the S-3CLpro as reported in a similar study.^12^

**Fig. 3:**
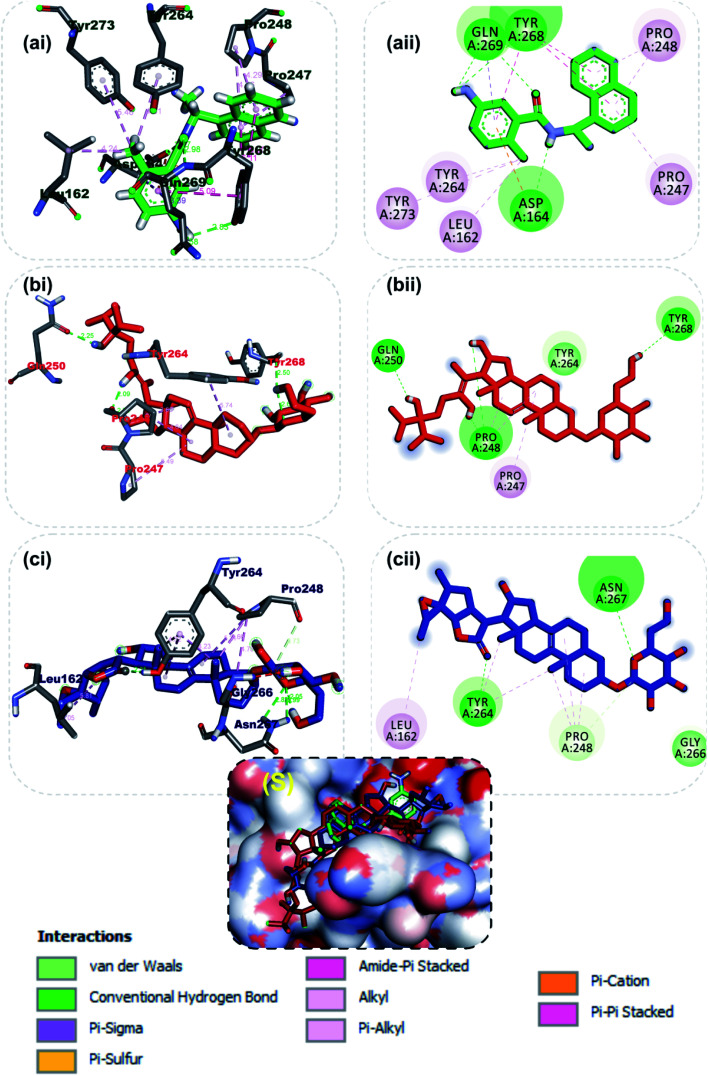
3D (i) and 2D (ii) amino acid interactions of phytochemicals and reference inhibitors in substrate binding cavity of SARS-CoV-2 PLpro. (S) Surface representation. Stick representations of the phytocompounds are shown by colours (a) Green: GRL0617 (b) Red: vernonioside A4 (c) Blue: vernonioside A2.

At present there are vast information about the PLpro as therapeutic target for SARS-CoV-2, thus multiple crystal structure of the proteins have been solved and deposited.^58,65^ Inhibitors of the S-PL^pro^ are broadly classified as either a covalent or non-covalent inhibitor. The later type is most significantly and extensively studied. Importantly, the noncovalent inhibitors such as GRL0617 do not bind at the active site, but instead nearby, below the BL2. Residues from the palm and thumb subdomains take part in forming protein–ligand interactions.^66^ The reference compound (GRL0617), a naphthalene-based inhibitor for PLpro was found to interact with important amino acid residues of the S3 and S4 of the substrate binding pocket, though no interaction was observed with the catalytic triad residues (CYS^111^, HIS^272^ and ASP^286^). The amino group of 4-methylcyclohexanamine and the carbonyl oxygen of GRL0617 formed 2 hydrogen bonds with Gln269. The carbonyl oxygen formed a carbon hydrogen bond with TYR268, while Asp164 accepted a hydrogen atom from amino group in-between the rings. The methyl group of 4-methylcyclohexanamine made 3 alkyl interactions with Tyr264, Tyr273 and Leu162 while the naphthalene rings formed pi–alkyl and pi–pi-T-shaped with the remaining interacting residues (Fig. 3a). The 6-hydroxyl of the glycosyl moiety, the 16 and 24′-hydroxyl group of the steroid aglycon of vernonioside A4 formed a conventional type of hydrogen bond with Tyr268, Pro248 and Gln250 of S-PL^pro^. The ring B and C formed alkyl interaction with Pro247 and Pro248 of PLpro. The carbonyl oxygen of the glycosyl and on the steroid aglycon of vernonioside A2 interacted with Asn267and Tyr264. 18, 19 and 27-methyl groups on the aglycon interacted *via* alkyl interaction with Pro28 and Tyr264, Tyr264 and Leu162 of PLpro respectively (Fig. 3b). While the A and B rings formed pi–alkyl interaction to Pro248 of S-PL^pro^. The 2 and 3-hydroxyl group of the glycosyl unit of vernoniamyoside C interacted *via* hydrogen bond to Try264 and Gln250 of S-PL^pro^ respectively, the 3-O-linkage between the aglycon and the glycosyl and 16-hydroxyl group formed a hydrogen bond with Asn267 and Asp164 of PLpro respectively. The B ring of the aglycon formed 2 pi–alkyl interactions with Pro247 and Pro248 of S-PL^pro^ respectively. Over all from our binding mode analysis, the lead phytochemicals adopt poses analogical to those from crystals with complexes of S-PL^pro^ with noncovalent inhibitors. Our results show that there are several residues that stand out in the frequency of forming relevant interactions with non-covalent phytochemicals. The most significant ones include Asp164, Tyr264, Tyr268, Pro247, and Pro248.

### 3.2 Optimization of docking interactions of steroidal saponins with SARS-CoV-2 3CL^Pro^ and PLpro representative conformations from MD simulation trajectories

**Fig. 4:**
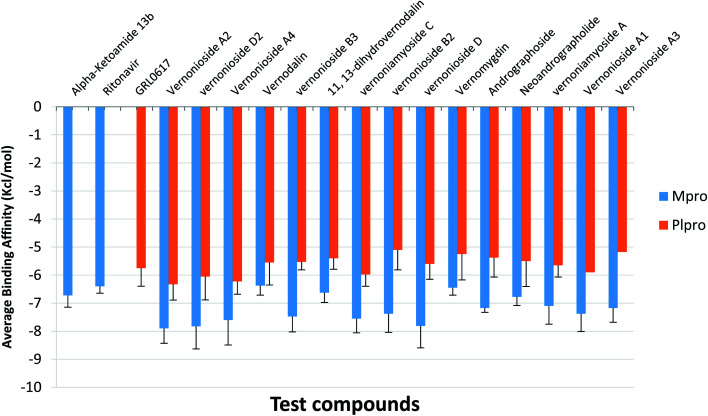
The mean binding energies and SEM of the reference inhibitors (ritonavir and α-ketoamide) and the ten lead phytochemicals against representative conformation obtained from the cluster analysis of the MD simulation trajectories of SARS-CoV-2 3CLpro and PLpro. Number of clusters: SARS-CoV-2 3CLpro = 4; PLpro = 4. The error bars represent the SEM (standard error of mean).

For an in-depth docking analysis to various representative conformers of the S-3CLpro and S-PLpro, the unbound proteins were subjected to 100 ns MD simulations. The thermodynamic parameters analysed for the apo proteins include SASA, RMSD, RoG and RMSF. The average values of SASA, RMSD, RoG and RMSF for S-3CLpro (Fig. S1 ESI†) are 27 800 Å^2^, 1.76 Å, 25.29 Å, and 1.06 Å, respectively, while S-PLpro (Fig. S2 ESI†) has average values of 28 516 Å^2^, 3.781 Å, 25.42 Å, and 1.38 Å, for SASA, RMSD, RoG, and RMSF, respectively. The TTClust cluster analysis of the MDS trajectories produced four clusters from the frame numbers listed in Table S3 (ESI).† From the four clusters for each protein a representative conformer was produced and used in the optimized docking analysis. The fifteen hit phytocompounds were docked into the active regions of the four different conformers of the target proteins. The average binding energies of the fifteen hit phytocompounds and the reference inhibitors to four different representative conformations is presented in Fig. 4. The binding energy values ranged from −7.175 kcal mol^−1^ (vernonioside A3) to −7.9 kcal mol^−1^ (vernonioside A2) for S-3CLpro, the that of S-PLpro ranged from −5.1 kcal mol^−1^ to −6.325 kcal mol^−1^. The optimized docking analysis further confirmed vernonioside A2 and vernonioside D2 to have the 2 best averaged binding energies to 3CL^pro^, while vernonioside A2 and vernonioside A4 had the 2 best averaged binding energies to PLpro. The best representative complexes of the two lead phytochemicals with a best docked pose and the highest binding energy to the best conformation was selected for MDS and interactive analysis using the PLIP webserver and Pymol. Representative structure from cluster 3 was selected as the best representative conformation of 3CL^pro^ and PLpro based on the observed binding energy with the lead phytochemicals. From the interaction analysis the most observed interactions types are hydrophobic contacts and few hydrogen bonding in some complexes. The most observed residues from the S-3CLpro that interacted with vernonioside A2 and vernonioside D2 (represented in bold in Table 4) are Pro168, Leu167, and Cys145, CYS^145^ is one of the S-3CLpro active site dyads (HIS^41^ and CYS^145^), and it was reported in all the ligands. The most observed residues from the PLpro that interacted with vernonioside A2 and vernonioside A4 are Asn109, Cys270, Ala107 His272, Trp106. His272 is one of the amino acid that forms the catalytic triad, while Trp106 are part of the amino acids at the catalytic region of S-PLpro (Table 4).

**Table 4:** Molecular interactions of selected steroidal saponins and reference inhibitors (α-ketoamide and ritonavir) with representative conformers of 3CL^pro^ and PLpro

SARS-CoV-2 protease	Compounds	Binding energies (kcal mol^−1^)	H-bonding		Hydrophobic interactions	
			Number	Residues from SARS-CoV-2 M^pro^	Number	Residues from SARS-CoV-2 M^pro^
3CL^pro^	Ritonavir	−6.4	4	Cys145, His41, Leu167, Glu166	2	Met165, Pro168
	Vernonioside A2	−6.3	5	Gly143, Ala191, Thr26	1	Pro168(2), Leu167, Cys145
	Vernonioside D2	−7.5	3	Arg188, Thr190 Pro168	1	Leu167, Met165, Cys145
PLpro	GRL0617	−6.9	3	Asn142, Gly271, Cys270	2	Asn109, and Trp106
	Vernonioside A2	−6.6	6	Trp106, Asn109, Gln269, Cys270, His272, Ans267	4	Lys94, Ala107, Trp106 (2)
	Vernonioside A4	−7.7	4	Gln269, Asn109, Cys270, Trp106	1	Lys92, Ala107, Trp93, Lys94, Lys105

### 3.3 Molecular dynamics simulations

**Fig. 5:**
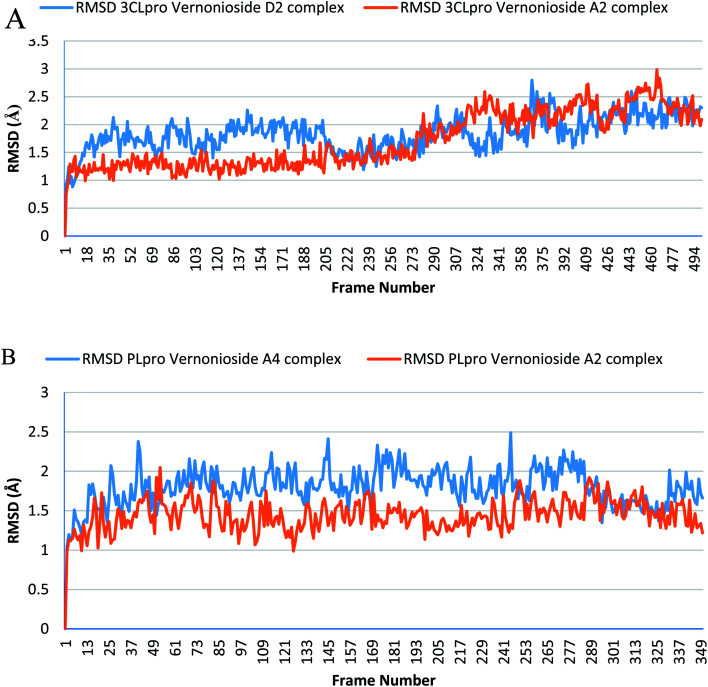
The root mean square deviation plots of selected complexes: (A) blue line: 3CLpro-vernonioside D2 and red line: 3CLpro-vernonioside A2; (B) PLpro-vernonioside A4 and PLpro-vernonioside A2.

**Fig. 6:**
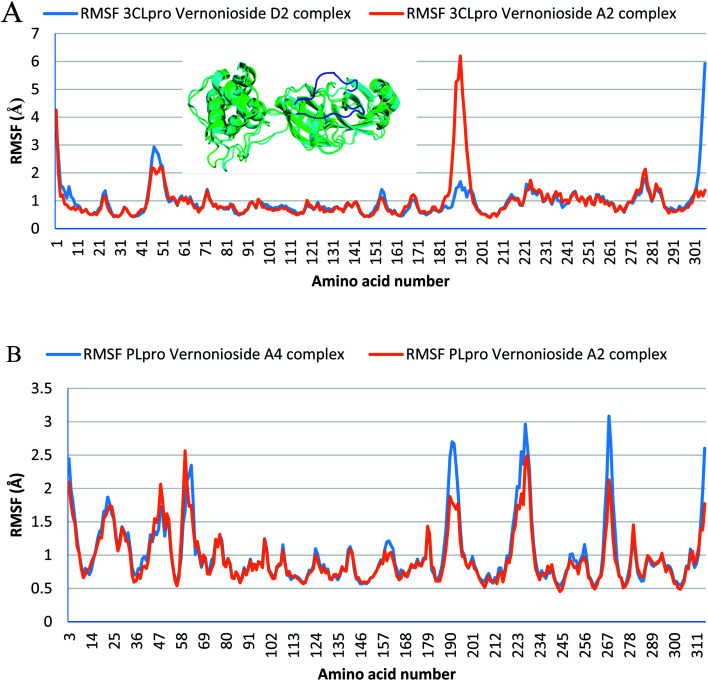
Per residue Root Mean Square Fluctuations (RMSF) plots selected complexes: (A) blue line: 3CLpro-vernonioside D2 and red line: 3CLpro-vernonioside A2; (B) PLpro-vernonioside A4 and PLpro-vernonioside A2. In (A) the alignment of frame 0 (green) and frame 500 (cyan) of 3CLpro-vernonioside A2 complex shows motion of the loop (blue) that caused a spike in the RMSF value.

**Fig. 7:**
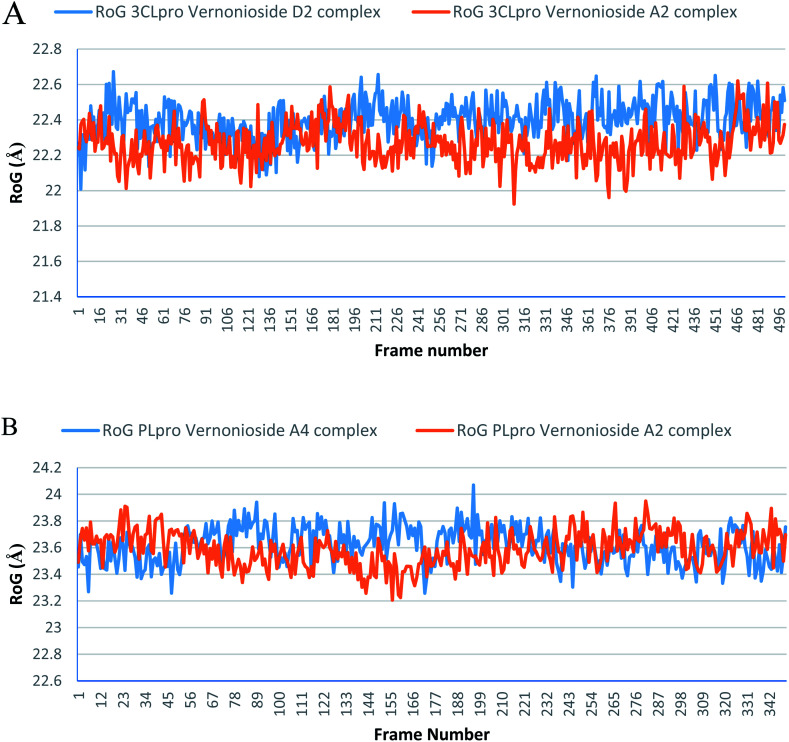
The radius of gyration (RoG) plots of selected complexes: (A) blue line: 3CLpro-vernonioside D2 and red line: 3CLpro-vernonioside A2; (B) PLpro-vernonioside A4 and PLpro-vernonioside A2.

**Fig. 8:**
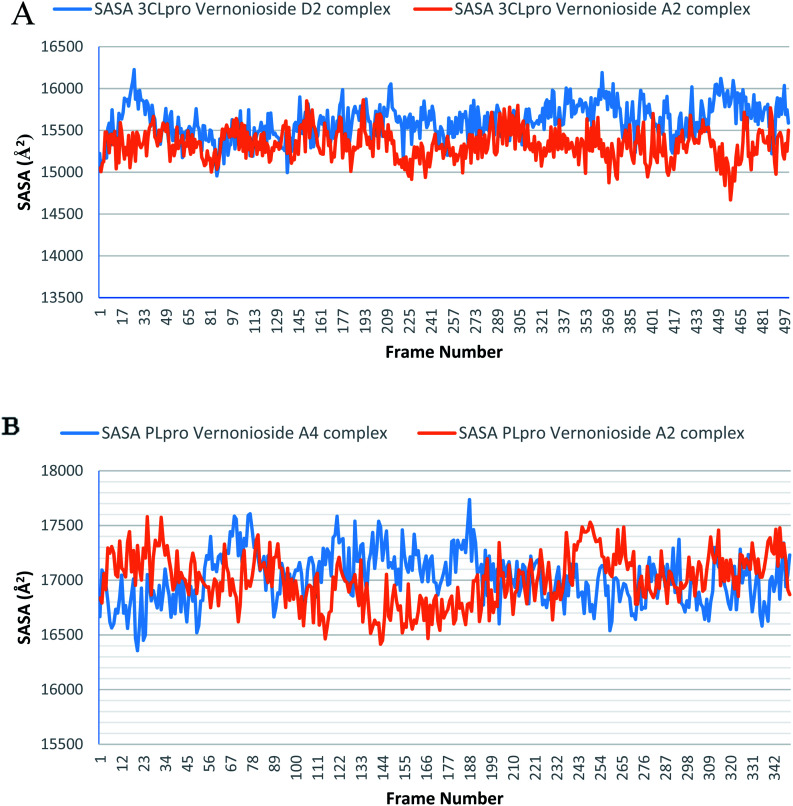
The Surface Accessible Surface Area (SASA) plots of selected complexes: (A) blue line: 3CLpro-vernonioside D2 and red line: 3CLpro-vernonioside A2; (B) PLpro-vernonioside A4 and PLpro-vernonioside A2.

**Fig. 9:**
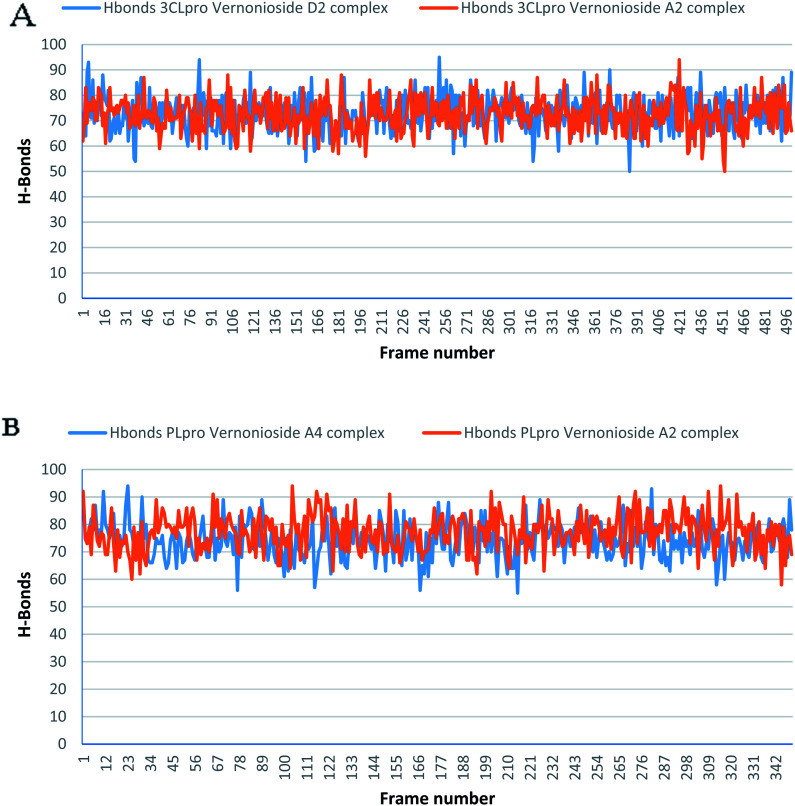
The changes in the number of H-bonds during the MDS trajectory of SARS-CoV-2 protease complexes: (A) blue line: 3CLpro-vernonioside D2 and red line: 3CLpro-vernonioside A2; (B) PLpro-vernonioside A4 and PLpro-vernonioside A2.

Monitoring the stability, structural/conformational fluctuations of the selected complexes of SARS-CoV-2 proteases and the two lead phytochemicals (S-3CLpro-vernonioside D2, S-3CLpro-vernonioside A2, S-PLpro-vernonioside A4 and S-PLpro-vernonioside A2) as compared with the apo (unbound) proteases was carried out in a simulated dynamic environment using the VMD version 1.9.3 and Tk console scripts. The RMSD is a plausible thermodynamic parameter of protein stability. RMSD plots show how much each frame is deviated from the initial conformation of the reference structure as a function of time. It is used to assess the differences between the structures sampled during the simulation and the reference structure.^67^Fig. 5A shows the RMSD for both S-3CLpro-vernonioside D2 and S-3CLpro-vernonioside A2 complexes. Both systems exhibited the same pattern with average values of 1.84 Å and 1.71 Å for S-3CLpro-vernonioside D2 and S-3CLpro-vernonioside A2 complexes, respectively. The RMSD values for S-PLpro-vernonioside A2 and S-PLpro-vernonioside A4 complexes are shown in Fig. 5B with an average values of 1.44 Å and 1.79 Å respectively. The binding of vernonioside A2 to the protease caused more compactness than vernonioside A4. The RMSF is another thermodynamic parameter that reveals the flexibility of different regions of a peptide chain and the amino acid residue along the trajectories, which can be related to crystallographic B factors.^67^ Residues with higher RMSF values connote greater fluctuations. Fig. 6A shows the RMSF plots for S-3CLpro-vernonioside D2 and S-3CLpro-vernonioside A2 complexes with average values of 0.96 Å and 0.99 Å, respectively. There are spikes occurring at the beginning and the end of the protein which correspond to the terminal motions. Alignment of the first and last frame explains the large values in RMSF for amino acids D187 to G195. RoG and SASA values give information about the folding of the protein. RMSF plots of S-PLpro-vernonioside A2 and S-PLpro-vernonioside A4 complexes (Fig. 6B) show approximately the same fluctuations with average values of 0.95 Å and 1.01 Å respectively. Adaptive variation in flexibility lies principally in the regions of the sequence that influence the conformational stabilities of the complexes.^68^ The radius of gyration was also monitored to evaluate the compactness of the bound structures. A stably folded protein maintains a reasonably steady RoG during the period of simulation. The stability of the complex is affected by loss of compactness through the introduction of weak intermolecular bonds.^69^ The SASA plots show the solvent accessibility surface of the proteins. Proteins with high RoG and SASA value indicates that the protein has undergone unfolding deviating from its original structure. Fig. 7 and 8 are plots for the RoG and SASA for S-3CLpro-vernonioside D2 and S-3CLpro-vernonioside A2 complexes respectively. An average values of 22.39 Å, 15 611 Å^2^ and 22.26 Å, 15 328 Å^2^, respectively was calculated from the plots. Both plots show a stable pattern for RoG and SASA values, which are emphasized through the stable number of H-bonds around average values of 72 H-bonds for both complexes (Fig. 9A). The average values of RoG and SASA for S-PLpro-vernonioside A2 and S-PLpro-vernonioside A4 complexes are 23.58 Å, 17 004 Å^2^ and 23.61 Å, 17 019 Å^2^, respectively. The plots for both systems showed the same behavior with a slight decrease in both RoG and SASA for the first 10 ns before rising again for S-PLpro-vernonioside A2 complex while the PLpro vernonioside A4 complex show a small increase in the first 9 ns before reaching a nearly steady pattern (Fig. 6B and 7B). The hydrogen bonds plots (Fig. 9B) show an average value of 77 and 74 bonds for S-PLpro-vernonioside A2 and S-PLpro-vernonioside A4 complexes, respectively. This further establishes a stable intramolecular backbone H-bonds among the residues upon the binding of the phytochemicals to the proteases.^69^

### 3.4 Molecular mechanics generalized Born surface area (MMGBSA) analysis

**Fig. 10:**
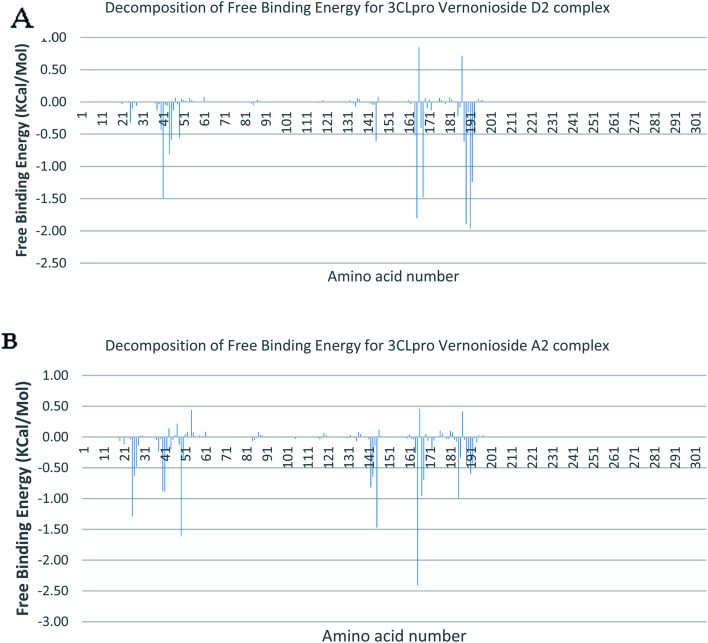
Molecular mechanics/Poisson–Boltzmann surface area (MM/PBSA) plot of binding free energy contribution per residue of SARS-CoV-2 3CLpro-phytochemical complexes (A) 3CLpro-vernonioside A2 (B) 3CLpro-vernonioside D2.

**Fig. 11:**
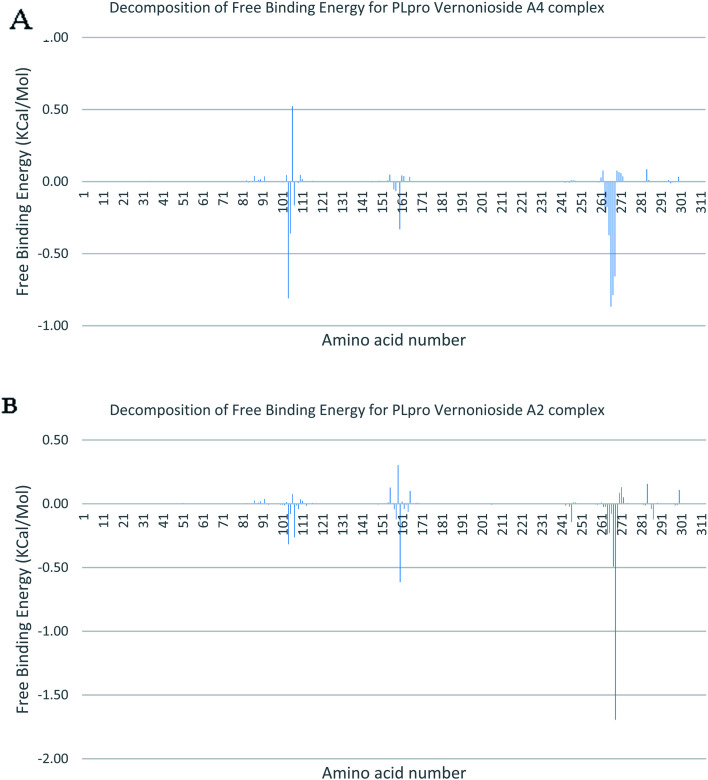
Molecular mechanics/Poisson–Boltzmann surface area (MM/PBSA) plot of binding free energy contribution per residue of SARS-CoV-2 PLpro complexes (A) PLpro-vernonioside A4 (B) PLpro-vernonioside A2.

In a dynamic environment, a quantitative simulation-based methods such as MMGB/PBSA provides substantially more accurate and reliable estimates of the binding affinity of a ligand to a receptor.^70^ It is computed based on the total binding free energy of the ligand–receptor complex. This involves the binding free energy (Δ*G*_bind_), which measures the affinity of a ligand to its target protein as well as the free energy difference between the ligand-bound state (complex) and the corresponding unbound states of proteins. Thus, the Δ*G*_bind_ calculations are important to gaining an in-depth knowledge about the binding modes of drug leads at the preliminary stages of drug design.^71^ In the current study, the MMGBSA free binding energy for S-3CLpro-vernonioside D2 and S-3CLpro-vernonioside A2 complexes were −24.55 kcal mol^−1^ and −25.38 kcal mol^−1^ respectively while the S-PLpro-vernonioside A2 and S-PLpro-vernonioside A4 complexes recorded a binding free energy of −9.96 kcal mol^−1^ and −7.82 kcal mol^−1^ respectively. The binding free energy calculations corroborated the static docking calculations. From the plots analysis, the most important amino acids for the binding of S-3CLpro to vernonioside A2 are T25, T26, R40, H41, M49, N142, G143, C145, M165, L167, P168, F185, A191 while for vernonioside D2 are H41, C44, T45, M49, C145, H164, M165, P168,R188, Q189, A191, and Q192 (Fig. 10A and B). For the two S-PLpro system, the amino acids that participated in the interaction between PLpro and vernonioside A2 are L162 and C270, while vernonioside A4 was stabilized in the dynamic state by interacting with W106, Y268, Q269, C270 of S-PLpro (Fig. 11A and B).

### 3.5 Clustering analysis of molecular dynamics simulation trajectories of SARS-CoV-2 3CLpro and PLpro complexes

The interactions in S-3CLpro and S-PLpro complexed with the dock phytochemicals were further studied through clustering analysis of the trajectory files. Table 5 shows the number and type of interactions between SARS-CoV-2 proteases and the docked phytochemicals. In the analysed clusters of S-3CLpro-phytochemicals system, interactions of the selected steroidal saponins with the catalytic residues of S-3CLpro were preserved while the S-PLpro-phytochemicals presented a reduced interaction. The clustering analysis further corroborates the observation of the MMGBSA binding free energy per-residue decomposition analysis.

**Table 5:** The cluster number and types of interactions for the SARS-CoV-2 proteases in complex with the two lead phytochemicals^a^

Protein–compound complex	Cluster	Hydrophobic interactions		H-bonds		Salt bridges	
		Number of interactions	Residues involved in the interaction	Number of interactions	Residues involved in the interaction	Number of salt bridges	Residues involved in the interaction
3CLpro–**vernonioside A2**	Cluster 1	6	T26 (3)–H41–N142–G143	3	H41–M165–P168		
	Cluster 2	5	T26 (2)–G143–V186–Q192	3	L50–L167–P168		
	Cluster 3	7	T26 (3)–H41–N119–N142–G143	1	M49		
	Cluster 4	3	T26–H41–E166	2	L50–M165		
3CLpro–**vernonioside D2**	Cluster 1	9	C44–N142 (2)–R188–T190 (2)–Q192 (3)	3	L27–M165–Q192	0	None
	Cluster 2	5	T25 (2)–S46–H164–Q192	1	A191	1	H41
	Cluster 3	3	H41–T45–Q192	3	M165–P168–Q189	1	H41
PLpro–**vernonioside A4**	Cluster 1	2	D108–N267	1	W106		
	Cluster 2	0	None	0	None		
	Cluster 3	0	None	0	None		
	Cluster 4	2	T158–G271	2	L162–Y264		
	Cluster 5	1	G271	0	None		
PLpro–**vernonioside A2**	Cluster 1	0	None	2	K92–A107		
	Cluster 2	0	None	1	Y268		
	Cluster 3	1	W106	0	None		

a Amino acid residues are represented in one-letter format and most common amino acids are in bold font.

### 3.6 *In silico* drug-likeness and pharmacokinetic characteristics of lead steroidal saponins

One of the most significant challenges in the drug design and development processes is the potential toxicity of the drug candidates. Although, the bioactive compounds in this study are known to be an integral of human diet and are assumed to be safe, it was necessary to check the toxicity and druggability. For this purpose, computational techniques, which are becoming increasingly useful for rapidity, simplicity and economic feasibility^72^ were employed. The results of the predictive drug-likeness and ADMET (Absorption, Distribution, Metabolism, Excretion, and Toxicity) filtering analyses of the three lead phytochemicals (TTRP) (vernonioside A2, vernonioside D2, and d vernonioside A4) obtained from the series of our *in silico* techniques ranging from the docking analysis to the cluster analyses MDS trajectories of SARS-CoV-2 3CL^pro^ and PLpro is represented in the Table 6. From these phytochemicals, vernonioside A2, and vernonioside D2, fulfilled at least four out of five basic requirements of the druglikeness parameters from the Lipinski rule of five that states that an orally active drug must not have more than one violation of the following criteria (not >5 H-bonds donors, not >10 H-bonds acceptors molecular mass not >500 daltons, octanol–water partition coefficient (log *P*) not >5). This suggests their favourable druggable properties (Table 6).^73^ The 3 steroidal saponins presented high gastrointestinal absorption, thereby suggesting high bioavailability.^74^ The permeability glycoprotein (P-gp) is widely expressed in the liver cells, epithelia of the intestine, cells of the kidney and the endothelial cells of the capillary comprising the blood–brain barrier and blood–testis barrier. On these surfaces it major function include pumping back xenobiotics into the intestinal lumen, urine-conducting ducts and capillaries^75^ (Lin & Yamazaki, 2003). The TTRP were non-substrate of the P-gp, these will further increases there bioavailability and reduce the rate of excretion which will in turn increase their half-lives.^75^ The TTRP presented low ability to cross the blood–brain barrier, probably due to their high molecular weight,^76^ this may prevent the compounds from getting to the brain cells that have been reported to be infected by SARS-CoV-2.^77^ The hERG channel plays a critical role in the repolarization and termination stages of action potential in cardiac cells, compounds that block the hERG channel may cause cardiotoxicity.^78^ The TTRP presented low potential of blocking the hERG channel, thereby suggesting that they may not cause hERG channel-related cardiotoxicity.^78^ The predictive analysis further revealed that the TTRP were not Ames mutagenic, human hepatotoxic, will not induce drug liver injury and skin sensitization, thereby dissociating them the ability to cause genetic mutations which, in turn, may initiate the pathophysiology of other diseases, such as cancer, hepatoxicity and adverse effect on the hepatocytes.^79,80^ The potential influence of the compounds on phase I drug metabolizing enzymes in the liver was also analysed using the various cytochrome P450 descriptors, the TTRP did not indicate adverse effects on phase I drug metabolism in the liver.^81,82^ The half life time and clearance rate of the TTRP were within acceptable range.

**Table 6:** Predictive physicochemical properties and ADMET^a^ parameters of the two lead phytocompounds to SARS-CoV-2 3CL^pro^ and PLpro

(a) Druglikeness properties	Vernonioside A2	Vernonioside A4		Vernonioside D2
Molecular weight (g mol^−1^)	632.35	648.8		648.78
Num. heavy atoms	46	47		46
Num. arom. heavy atoms	0	0		0
Num. rotatable bonds	7	8		6
Num. H-bond acceptors	10	11		10
Hydrogen bond donor	5	7		4
cLog *P*	2.14	0.93		2.64
Molar refractivity	169.29	172.17		166.66
TPSA (Å^2^)	158.44	186.37		147.44

**Drug-likeness**				
Lipinski	Yes	No		Yes
Bioavailability score	0.55	0.17		0.56

**Absorption (probability)**				
(b) Admet SAR				
HIA	HIA + (0.961)	HIA + (0.960)		HIA + (0.960)
Caco-2 permeability *C* m s^−1^	Caco2 + (−5.772)	Caco2 + (−0.84)		Caco2 + (−5.661)
P-glycoprotein substrate	Negative (0.826)	Negative (0.778)		Negative (0.88)

**Distribution (probability)**				
Blood–brain barrier	BBB − (0.123)	BBB − (0.476)		BBB − (0.34)
PPB%	69.685	67.689		79.737
VD L kg^−1^	−0.322	−0.142		−0.186

**Metabolism (probability)**				
CYP450 1A2 inhibitor	Negative (0.047)	Negative (0.039)		Negative (0.055)
CYP450 1A2 substrate	Negative (0.318)	Negative (0.297)		Negative (0.297)
CYP450 3A4 inhibitor	Negative (0.457)	Negative (0.515)		Positive (0.592)
CYP4502C9 inhibitor	Negative (0.315)	Negative (0.365)		Negative (0.183)
CYP450 2C9 substrate	Negative (0.288)	Negative (0.296)		Negative (0.372)
CYP4502C19 inhibitor	Negative (0.072)	Negative (0.009)		Negative (0.111)
CYP450 2C19 substrate	Negative (0.384)	Negative (0.324)		Negative (0.35)
CYP4502D6 inhibitor	Negative (0.298)	Negative (0.295)		Negative (0.32)
CYP450 2D6 substrate	Negative (0.223)	Negative (0.238)		Negative (0.253)
**Elimination**				
*T*_1/2_ (half life time)	2.292 h	2.302 h		2.388 h
CL (clearance rate) mL min^−1^ kg^−1^	0.972	1.055		1.04

**Toxicity**				
hERG blockers	Negative (0.256)		Negative (0.474)	Negative (0.101)
H-HT	Negative (0.428)		Negative (0.468)	Negative (0.41)
AMES	Negative (0.312)		Negative (0.252)	Negative (0.312)
SkinSen	Negative (0.286)		Negative (0.259)	Negative (0.286)
LD_50_ (LD_50_ of acute toxicity)	4.22 −log mol kg^−1^ (38.975 mg kg^−1^)		4.36 −log mol kg^−1^ (28.933 mg kg^−1^)	4.282 −log mol kg^−1^ (33.02 mg kg^−1^)
DILI	Negative 0.154		Negative (0.136)	Negative 0.154

a ADMET: absorption, distribution, metabolism, P-gp: permeability glycoprotein; GI: gastro-intestinal; BBB: blood–brain barrier; CYP: cytochrome P450; hERG: human ether-à-go-go-related gene; HIA: human intestinal absorption; H-HT: human hepatotoxicity AMES: Ames mutagenicity; DILI: drug induced liver injury; VD: volume distribution; PPB: plasma protein binding. (Values in bracket represent the probability.) The acceptable range for each descriptor is on (https://admet.scbdd.com/calcpre/index_sys_result/).

### 3.7 Pharmacophore features of lead phytochemicals

**Fig. 12:**
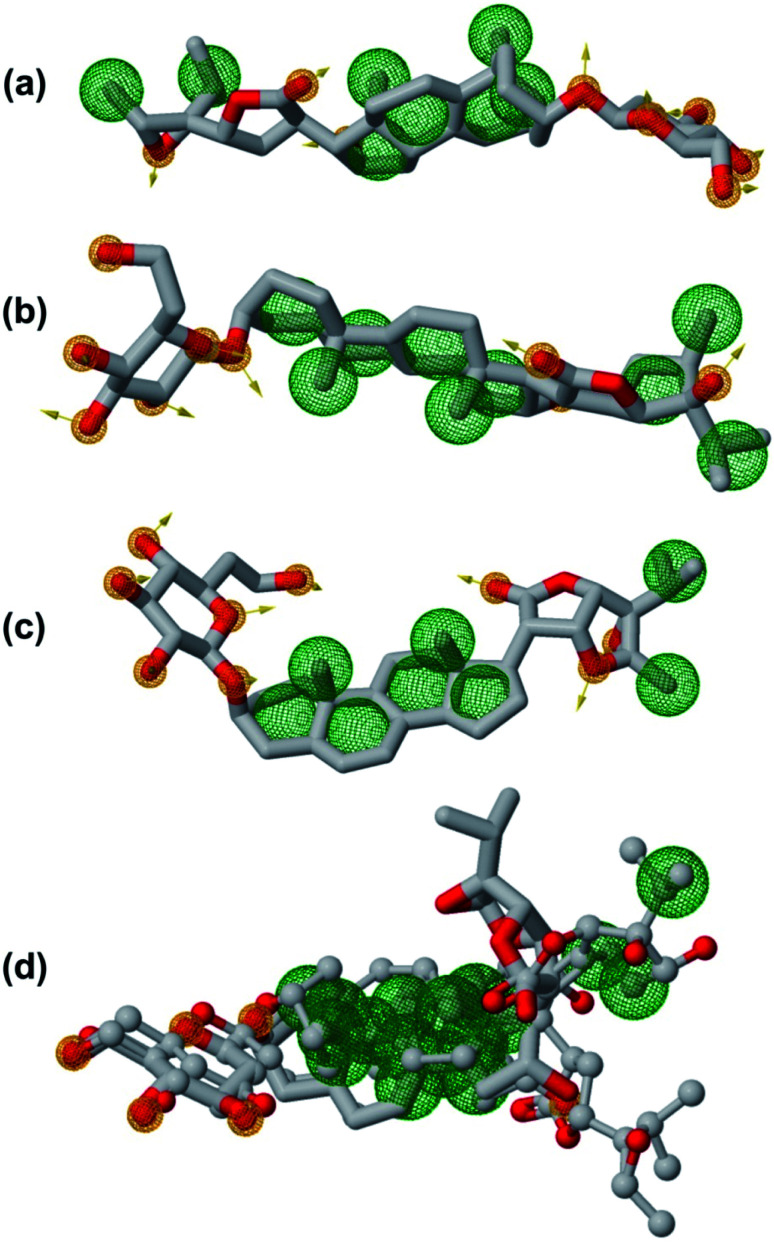
The pharmacophoric features of lead phytochemicals. (a) Vernonioside A2 (b) vernonioside A4 (c) vernonioside D2 (d) merged pharmacophore of the three phytochemical. Round meshes represent pharmacophoric features. Orange – hydrogen bond acceptor and hydrogen bond donor and green – hydrophobic.

In order to generate a pharmacophore model that describes characteristic features of the lead phytochemicals were subjected to pharmacophoric investigated. Table 7 and Fig. 12 show the details of generated pharmacophores from the lead phytochemicals (vernonioside A2 and vernonioside A4 and vernonioside D2). The generated pharmacophores for the individual phytochemical were aligned and paired. Table 8 shows the pharmacophoric features of the aligned lead phytochemicals and best pairs. The pharmacophore, having the highest score and maximum features were selected. Vernonioside A2 and vernonioside D2 were selected as the best pairs for the SARS-COV-2 3CL^pro^, while vernonioside A2 and vernonioside A4 were selected for SARS-COV-2 PL^pro^. Comparing the pharmacophore of the individual lead phytochemicals (Fig. 12a–c) and merged pharmacophore model (Fig. 12d), the later provided a larger coverage of potential features that can be exploited for further screening studies on independent databases. The importance of these features in the binding to of catalytic residues of the 3CL^pro^ has been reported in previous studies to possess a vectorial nature which indicates the direction of electron sharing.^83,84^

**Table 7:** Pharmacophoric features of the lead phytochemicals^a^

Atoms	Features	Spatial features	Aromatic	Hydrophobic	Donors	Acceptors	Negatives	Positives
V. A2	96	38	28	0	25	4	9	0
V. A4	100	43	26	0	27	6	10	0
V. D2	92	36	28	0	22	4	10	0

a V. – vernonioside.

**Table 8:** Pharmacophoric features of best pair wise solution of lead phytochemicals^a^

Compounds	Scores	Features	Spatial features	Aromatic	Hydrophobic	Donors	Acceptors	Negatives	Positives
V. A2, V. A4, and V. D2	35.916	22	19	0	13	3	6	0	0
V. A2 and V. D2	22.75	27	23	0	16	4	7	0	0
V. A2 and V. A4	19.66	19	16	0	9	3	7	0	0

a V. – vernonioside.

## 4 Conclusion

The life-threatening COVID-19 continues its surge across the globe, despite the development of vaccines. The viral cysteine proteases (3CL^pro^ and PL^pro^), which are critical enzymes that control SARS-CoV-2 replication and infectivity are attractive therapeutic targets in COVID-19. Here in, we employed structure-based screening methods to screen 176 phytochemicals from four West African antiviral culinary herbs and spices, *viz*: African tea leaf (*Vernonia amygdalina*), African basil (*Ocimum gratissimum*), *Aframomum melegueta*, and *Piper guineense*, for inhibitors that may simultaneously target the SARS-CoV-2 cysteine proteases (3CLpro and PLpro). Three steroidal saponins from *Vernonia amygdalina* (vernonioside A2, vernonioside D2 and vernonioside A4) demonstrated high binding tendencies with the representative conformation obtained from the cluster analysis molecular dynamics simulation (MDS) trajectories files of the 3CL^pro^ and PLpro of SARS-CoV-2. These compounds were observed to be within the active sites, and interacted with the catalytic the catalytic residues thereby exhibiting dual-targeted inhibitory potential. The binding free energy calculation base on Molecular Mechanics Generalized Born Surface Area (MM-GBSA) corroborated the static docking analysis. The MD simulation of the complex systems demonstrated high stability. The three steroidal saponins fulfilled the basic requirements for various drug-likeness and ADMET descriptors thereby suggesting their druggability. Therefore they are recommended for further *in vitro* and *in vivo* studies for drugs against coronavirus diseases.

## Authors contributions

Conceptualization, G. A. G.; visualization, G. A. G.; original draft preparation, G. A. G. and O. M. O.; methodology, G. A. G. and O. M. O; data curation: M. M. F. and J. O. O.; software, G. A. G., O. M. O. and I. M. I.; writing-review & editing G. A. G. and O. M. O; supervision, J. O. A. and C. O. O.

## Conflicts of interest

The authors declare that they have no competing interests.

## Supplementary Material

RA-011-D1RA05976A-s001
