# Supplementary material for: Dietary stigmastane-type saponins as promising dual-target directed inhibitors of SARS-CoV-2 proteases: a structure-based screening
Source: RSC Adv. 2021 Oct 12;11(53):33380–98. doi: 10.1039/d1ra05976a (PMC9042289; doi:10.1039/d1ra05976a)
Supplement: RA-011-D1RA05976A-s001 [file RA-011-D1RA05976A-s001.pdf]

Electronic Supplementary Material (ESI) for RSC Advances.
This journal is © The Royal Society of Chemistry 2021

Supplementary Data

Dietary stigmastane-type saponins as promising dual-target directed inhibitors of SARS-CoV-2 proteases: a structure-based screening

Oludare M. Ogunyemi[1,4], Gideon A. Gyebi[2], Ibrahim M. Ibrahim[3], Charles O. Olaiya[4], Joshua O. Ocheje[5], Modupe M. Fabusiwa[1] Joseph O. Adebayo[6]

[1]Human Nutraceuticals and Bioinformatics Research Unit, Department of Biochemistry, Salem University, Lokoja, Nigeria

[2]Department of Biochemistry, Faculty of Science and Technology Bingham University, Karu, Nasarawa, Nigeria

[3]Department of Biophysics, Faculty of Sciences, Cairo University, Giza Egypt

[4]Nutritional and Industrial Biochemistry Unit, Department of Biochemistry, University of Ibadan, Nigeria;

[5]Department of Pure and Industrial Chemistry, Nnamdi Azikiwe University, Akwa, Nigeria

[6]Department of Biochemistry, Faculty of Life Sciences, University of Ilorin, Ilorin, Nigeria

***Corresponding Author**
Gideon Ampoma GYEBI
Email: gideonagyebi@gmail.com/gyebi.gideon@binghamuni.edu.ng
Tel: +2347063983652
Address:
Department of Biochemistry, Faculty of Science and Technology
P.M.B 005, Karu, Nasarawa State, Nigeria

Orcid ID: 0000-0002-1945-1739

Table S1: Binding energies of phytochemicals docked in the active sites of SARS-CoV-2 3-Chymotrypsin-Like protein

S/N	Compounds	**Binding energies (Kcal/mol)**
S1	Alpha-Ketoamide 13b	-7.7
S2	Ritonavir	-6.8
S3	10659	-5.3
1	Vernonioside A22	-8.6
2	vernonioside D22	-8.4
3	Vernonioside A42	-8.3
4	Vernodalin	-8.1
5	vernonioside B32	-8.1
6	11, 13-dihydrovernodalin	-8.0
7	vernoniamyoside C2	-8.0
8	vernonioside B22	-8.0
9	vernonioside D2	-8.0
10	Vernomygdin	-7.9
11	Andrographoside	-7.8
12	Neoandrographolide	-7.8
13	vernoniamyoside A2	-7.8
14	Vernonioside A12	-7.8
15	Vernonioside A32	-7.8
16	Veronicoside	-7.8
17	3,7,8-trimethoxy-1-hydroxy-xanthone2	-7.7
18	7-O-methylwogonin-5-glucoside2	-7.7
19	Basilimoside	-7.7
20	vernodalinol2	-7.7
21	vernoniamyoside D2	-7.7
22	1,2-dihydroxy-6, 8-dimethoxy-xanthone2	-7.6
23	1,2,2,3-tetrahydroovernoodalin2	-7.5
24	Andrographidoid C2	-7.5
25	Hydroxyvernolide	-7.5
26	Vernomygdin2.	-7.5
27	Andrographidoid D2	-7.4
28	chicoric acid	-7.4
29	Luteolin	-7.4
30	Myricetin	-7.4
31	Vernolide	-7.4
32	vernoniamyoside B2	-7.4
33	14-deoxy-11, 12-didehydroandrographolide	-7.3
34	Andrographidoid A2	-7.3
35	Dihydroskullcapflavone	-7.3
36	Nepetoidin A	-7.3
37	Vernodalol	-7.3

38	vernonioside B12	-7.3
39	5-hydroxy-7, 2, 3-trimethoxy flavone2	-7.2
40	5-hydroxy-7, 8, 2, 3-tetramethoxyflavone2	-7.2
41	Hymenoxin	-7.2
42	luteolin 7-O-glucoside2.	-7.2
43	1, 8-dihydroxy-3, 7-dimethoxy-xanthone2	-7.1
44	5, 7, 2, 3-tetramethoxyflavonone2	-7.1
45	5-hydroxy-7,2,6-trimethoxyflavone2	-7.1
46	7-O-methyldihydrowogonin	-7.1
47	Andrographidoid B2	-7.1
48	Nevadensin	-7.1
49	Salvigenin	-7.1
50	vernonioside E2	-7.1
51	1,2,3,15,11,13,2,3-octahydrovernodalin2	-7.0
52	1,2,11,13,2,3-hexahydrovernodalin2.	-7.0
53	4-deoxyandrographolide	-7.0
54	7-O-methylwogonin	-7.0
55	14-deoxy-15-isopropylidene-11,12-didehydroandrographolide	-7.0
56	Andrograpanin	-7.0
57	Andrographolactone	-7.0
58	Andrographolide2	-7.0
59	isohamnetin	-7.0
60	Rosmarinic acid	-7.0
61	Vernolepin	-7.0
62	Vernomenin	-7.0
63	Xanthomicrol	-7.0
64	Apigenin 7,4'-dimethyl ether	-6.9
65	Beta-Sitosterol	-6.8
66	4,8-dihydroxy-2,7-dimethoxy-xanthone2	-6.7
67	4,15-dihydrovernodalin2	-6.7
68	DOG2	-6.5
69	Epivernodalol2	-6.5
70	CAT2	-6.3
71	beta_caryophyllene epoxide	-6.2
72	humulene oxide	-6.1
73	Caryophyllene oxide	-6.0
74	beta_eusdemole	-5.9
75	alloaromadendrene	-5.8
76	alpha-Humulene	-5.8
77	cubebol	-5.8
78	Germacrene D	-5.8
79	Guaiazulene2	-5.8

80	Sinapic acid	-5.8
81	Spathulenol	-5.8
82	4 epi cubebol	-5.7
83	alpha muurolol	-5.7
84	alphaCopaene	-5.7
85	Aromadendrene	-5.7
86	Caryophyllene	-5.7
87	epi cubenol	-5.7
88	muurolol2	-5.7
89	ylangene	-5.7
90	Andrographidoid E2	-5.6
91	chamigrene	-5.6
92	isolongifolol	-5.6
93	alpha Calacorene2	-5.5
94	alphaCubebene	-5.5
95	alpha-Muurolene	-5.5
96	beta- Copaene	-5.5
97	Beta_Cubebene	-5.5
98	Bicyclogermacrene	-5.5
99	calamenene	-5.5
100	gamma_Cadinene	-5.5
101	gamma-Cadinene	-5.5
102	iso-eugenol	-5.5
103	longifoline	-5.5
104	beta- Bourbonene	-5.4
105	beta -Selinene2	-5.4
106	Elemol	-5.3
107	Vernolic	-5.3
108	bisabolol	-5.2
109	Carvacrol	-5.2
110	cis-Sabinene hydrate	-5.2
111	Methyl cinnamate	-5.2
112	alpha_Bergamotene	-5.1
113	alpha-Elemene2	-5.1
114	Beta_Elemene	-5.1
115	Isophytol2	-5.1
116	linoleic2	-5.1
117	Thymol acetate	-5.1
118	Beta_bisabolene	-5.0
119	pinocarveol2	-5.0
120	Eugenol	-4.9
121	linalyl acetate	-4.9
122	Thymol	-4.9
123	Vanillin	-4.9
124	1,8-Cineole	-4.8
125	carvone	-4.8
126	eucalyptol.	-4.8
127	methyl eugenol	-4.8

128	Methyl salicylate2	-4.8
129	beta -Thujone2	-4.7
130	carveol	-4.7
131	isopinocamphone	-4.7
132	nerolidol	-4.7
133	pCymen-8-ol	-4.7
134	Pinocarvone	-4.7
135	Terpinen-4-ol	-4.7
136	Thymol methyl ether	-4.7
137	alpha-Terpineol	-4.6
138	fenchone	-4.6
139	myrtanal	-4.6
140	Neoisomenthol	-4.6
141	trans-pinocarveol	-4.6
142	trans-Thujone	-4.6
143	alpha-Terpinene	-4.5
144	Beta_Phellandrene	-4.5
145	Borneol	-4.5
146	Farnesene	-4.5
147	gamma-3-Carene	-4.5
148	Nerol	-4.5
149	Terpinolene	-4.5
150	trans-Sabinene hydrate	-4.5
151	alpha-Thujene	-4.4
152	citronellol	-4.4
153	gamma-Terpinene2	-4.4
154	Linalool	-4.4
155	pCymene	-4.4
156	1,3,8-p-Menthatriene	-4.3
157	alpha-Phellandrene	-4.3
158	estragole	-4.3
159	gama-2-Carene	-4.3
160	Limonene	-4.3
161	O-cymene2	-4.3
162	pCymenene	-4.3
163	P-Tolylpropene	-4.3
164	Beta_Pinene	-4.2
165	Pinene	-4.2
166	alpha-Pinene	-4.1
167	Sabinene	-4.1
168	Camphene	-4.0
169	Zbeta Ocimene	-4.0
170	1-Octen-3-ol	-3.9
171	Ocimene	-3.9
172	Myrcene	-3.7
173	Hexenol	-3.4
174	Obatoclax	-3.4
175	cubenene	-3.3

176	Veronicoside A	-1.7

Table S2: Binding energies of phytochemicals docked in the active sites of 3-Chymotrypsin-like and Papain-like proteases of SARS-CoV-2

S/No	Phytochemicals	Binding energies (Kcal/mol)	
		SARS-CoV-2 3CLpro	SARS-CoV-2 PLpro
S1	Alpha-Ketoamide 13b	**-7.7**	
S2	Ritonavir	-6.8	
S3	GRL0617		-6.7
1	Vernonioside A22	**-8.6**	**-6.8**
2	vernonioside D22	**-8.4**	-6.3
3	Vernonioside A42	**-8.3**	**-7.2**
4	Vernodalin	-8.1	-5.9
5	vernonioside B32	-8.1	-6.1
6	11, 13-dihydrovernodalin	-8.0	-5.7
7	vernoniamyoside C2	-8.0	**-6.7**
8	vernonioside B22	-8.0	-5.9
9	vernonioside D2	-8.0	-6.1
10	Vernomygdin	-7.9	-5.3
11	Andrographoside	-7.8	-6.2
12	Neoandrographolide	-7.8	**-6.7**
13	vernoniamyoside A2	-7.8	-6.2
14	Vernonioside A12	-7.8	-6.2
15	Vernonioside A32	-7.8	-6.2
16	Veronicoside	-7.8	-6.5
17	3,7,8-trimethoxy-1-hydroxy-xanthone2	**-7.7**	-6.3
17	7-O-methylwogonin-5-glucoside2	**-7.7**	-6.3
18	Basilimoside	**-7.7**	**-6.7**
19	vernodalinol2	**-7.7**	-6.3
20	vernoniamyoside D2	**-7.7**	-6.0
21	1,2-dihydroxy-6, 8-dimethoxy-xanthone2	-7.6	-5.9
22	1,2,2,3-tetrahydroovernoodalin2	-7.5	-5.4
23	Andrographidoid C2	-7.5	-6.1
24	Hydroxyvernolide	-7.5	-5.7
25	Vernomygdin2.	-7.5	-5.9
26	Andrographidoid D2	-7.4	-6.2
27	chicoric acid	-7.4	-5.8
28	Luteolin	-7.4	-6.3
29	Myricetin	-7.4	-6.0
30	Vernolide	-7.4	-5.7
31	vernoniamyoside B2	-7.4	-6.4
32	14-deoxy-11, 12-didehydroandrographolide	-7.3	-6.0
33	Andrographidoid A2	-7.3	-5.9
34	Dihydroskullcapflavone	-7.3	-5.9
35	Nepetoidin A	-7.3	-6.0

36	Vernodalol	-7.3	-5.1
37	vernonioside B12	-7.3	-5.8
38	5-hydroxy-7, 2, 3-trimethoxy flavone2	-7.2	-5.6
39	5-hydroxy-7, 8, 2, 3- tetramethoxyflavone2	-7.2	-5.8
40	Hymenoxin	-7.2	-5.7
41	luteolin 7-O-glucoside2.	-7.2	-6.5
42	1, 8-dihydroxy-3, 7-dimethoxy-xanthone2	-7.1	-5.8
43	5, 7, 2, 3-tetramethoxyflavonone2	-7.1	-5.5
44	5-hydroxy-7,2,6-trimethoxyflavone2	-7.1	-5.3
45	7-O-methyldihydrowogonin	-7.1	-5.8
46	Andrographidoid B2	-7.1	-5.9
47	Nevadensin	-7.1	-5.8
48	Salvigenin	-7.1	-5.8
49	vernonioside E2	-7.1	-6.0
50	1,2,3,15,11,13,2,3-octahydrovernodalin2	-7.0	-5.2
51	1,2,11,13,2,3-hexahydrovernodalin2.	-7.0	-5.4
52	4-deoxyandrographolide	-7.0	-6.0
53	7-O-methylwogonin	-7.0	
54	14-deoxy-15-isopropylidene-11,12-didehydroandrographolide	-7.0	-6.4
55	Andrograpanin	-7.0	-5.7
56	Andrographolactone	-7.0	**-7.0**
57	Andrographolide2	-7.0	-6.0
58	isohamnetin	-7.0	-6.2
59	Rosmarinic acid	-7.0	-5.9
60	Vernolepin	-7.0	-5.8
61	Vernomenin	-7.0	-5.4
62	Xanthomicrol	-7.0	-5.6
63	Apigenin 7,4'-dimethyl ether	-6.9	-5.8
64	Beta-Sitosterol	-6.8	**-6.8**

Figures in bold represent the top three compounds in view of binding scores.

Table S3: Clusters obtained from the Clustering analysis of the MD simulation trajectories of unbound SARS-CoV-2 3CLpro and SARS-CoV-2 PLpro.

Cluster Number	SARS-CoV-2 3CLpro Frames	SARS-CoV-2 PLpro Frames
Cluster 1	f273-s511	f19-s86
Cluster 2	f638-s81	f796-s102
Cluster 3	f61-s158	f762-s251
Cluster 4	f919-s250	f154-s561

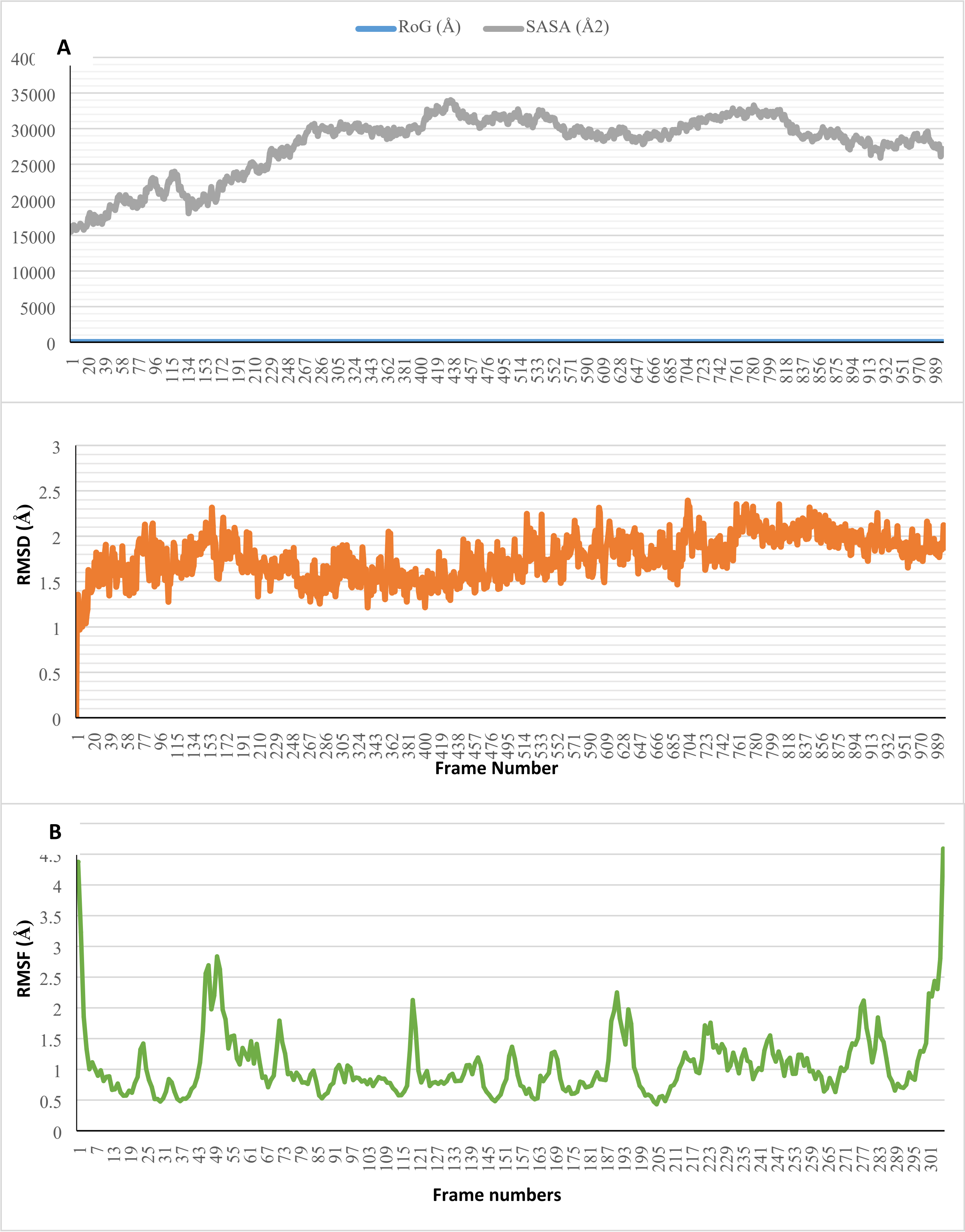

Figure S1: (A) The SASA (Grey line), RoG (Blue line); (B) the RMSD (Red line) and (C) RMSF (Green line) plots of molecular dynamics (MD) simulation of SARS-CoV-2 3CLpro.

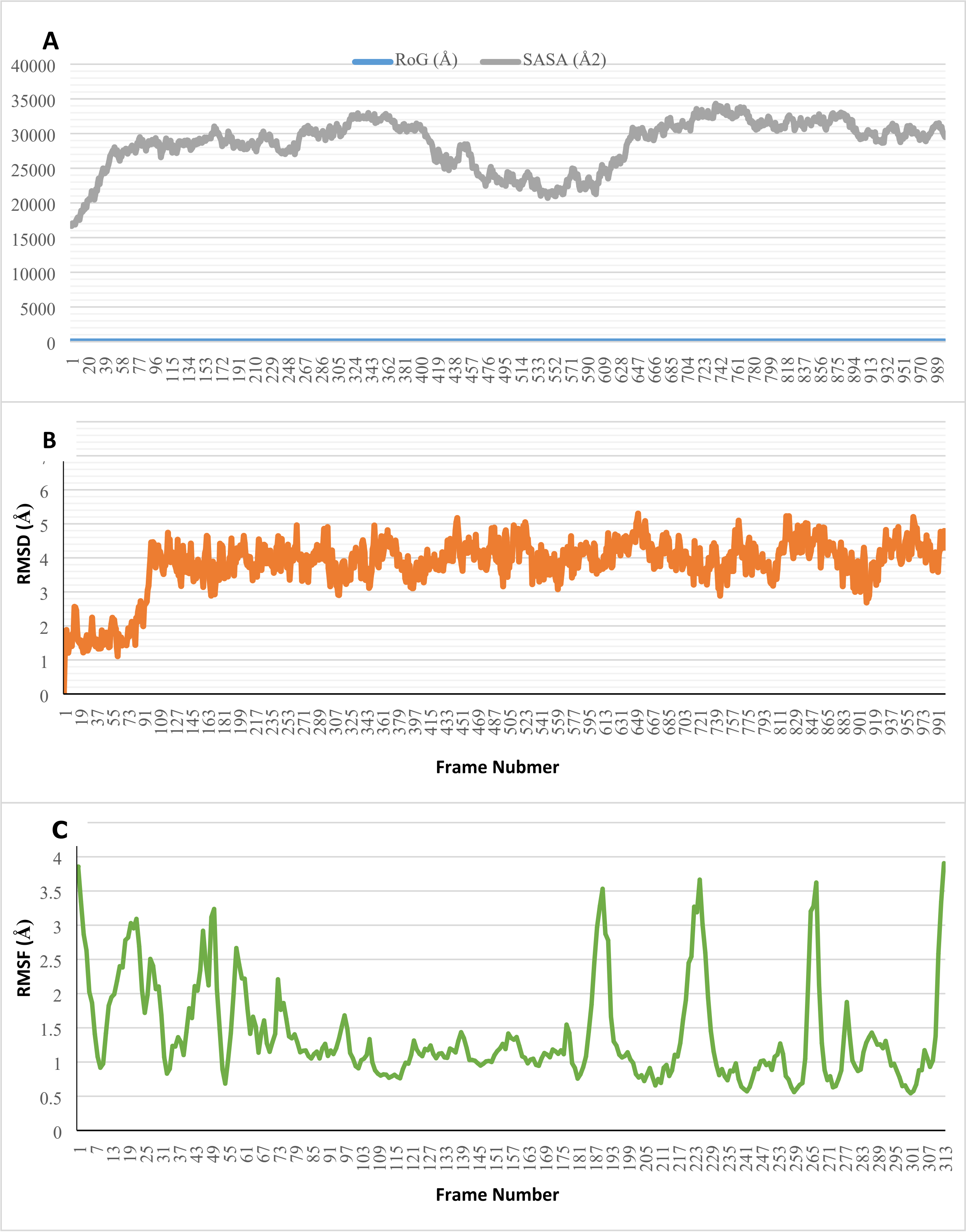

Figure S2: (A) The SASA (Grey line), RoG (Blue line); (B) the RMSD (Red line) and (C) RMSF (Green line) plots of molecular dynamics (MD) simulation of SARS-CoV-2 3CLpro.
